# Sociodemographic determinants, knowledge, and patterns of e-cigarette use among adults in Riyadh, Saudi Arabia: A cross-sectional study

**DOI:** 10.18332/tid/231135

**Published:** 2026-10-06

**Authors:** Bayan Alzahrani, Peter Cahusac, Abdullah Alsharif, Manal Alhumud, Noara Alhusseini

**Affiliations:** 1 Department of Biostatistics, Epidemiology and Public Health, College of Medicine, Alfaisal University, Riyadh, Saudi Arabia; 2 Department of Community Health Sciences, College of Applied Medical Sciences, King Saud University, Riyadh, Saudi Arabia

**Keywords:** electronic cigarettes, sociodemographic determinants, knowledge, patterns, Riyadh City

## Abstract

**INTRODUCTION:**

Electronic cigarette (e-cigarette) use has increased globally and in Saudi Arabia. Evidence regarding sociodemographic determinants, perceptions, and patterns of use among Saudi adults remains limited. This study investigated sociodemographic determinants, knowledge, perceptions, and patterns of e-cigarette use among Saudi adult users in Riyadh.

**METHODS:**

A cross-sectional study was conducted among 207 Saudi adult e-cigarette users residing in Riyadh from 21 January to 18 April 2024. Participants were recruited using a convenience sampling strategy. Data were collected using a self-administered online questionnaire distributed through social media platforms. The inclusion criteria included Saudi adults aged ≥18 years who reported current e-cigarette use and resided in Riyadh. Data were analyzed using jamovi (version 2.6). Descriptive statistics, likelihood ratio tests, correlation analyses, and regression models were used.

**RESULTS:**

Most participants were male (71.0%) and aged 18–39 years. Friends and relatives were the primary source of information about e-cigarettes (80%). Although awareness of the legality of e-cigarettes was high (85%), misconceptions remained common. Approximately 42.5% believed e-cigarettes were safer than conventional cigarettes, while 13% believed that only water vapor was emitted. Gender was significantly associated with vaping situations (Likelihood ratio χ^2^(3)=8.215, p=0.042), safety perceptions (LR χ^2^(2)=9.226, p=0.010), vaping frequency (B=1.870; 95% CI: 0.913–2.891; p<0.001), and harm perception (B= -1.583; 95% CI: -2.947 – -0.219; p=0.023), with males reporting higher vaping frequency and lower perceived harm. Participants who reported successful smoking cessation through vaping also perceived lower levels of harm (B= -2.802; 95% CI: -4.496 – -1.108; p=0.001).

**CONCLUSIONS:**

Gender, age, and current occupation were linked to vaping use, with males more likely to vape frequently, age was associated with vaping locations, and occupation was linked to vaping situations. Perceptions and knowledge were associated with demographic factors, revealing relationships between gender and safety and emissions beliefs, as well as age and legality and chemical awareness. Furthermore, individuals who quit tobacco through vaping and males generally had lower harm perceptions. Future studies should be conducted to capture these associations.

## INTRODUCTION

Electronic cigarettes (e-cigarettes), also known as electronic nicotine delivery systems (ENDS), have emerged as one of the most widely used alternative nicotine products worldwide^1^. Their popularity has increased substantially over the past decade, particularly among adolescents and young adults, largely because they are marketed as less harmful alternatives to conventional cigarettes and as potential smoking cessation aids^2^. Global estimates indicate that e-cigarette use continues to rise across many regions, with the number of users increasing from 58.1 million in 2018 to 68 million in 2020^3^. Despite increasing public acceptance, concerns remain regarding the health effects of long-term e-cigarette use and the potential for nicotine dependence among users^4^.

Although e-cigarettes do not involve tobacco combustion, they are not risk-free. E-cigarette aerosols contain nicotine, ultra-fine particles, volatile organic compounds, flavoring agents, and potentially toxic metals that may adversely affect respiratory and cardiovascular health^4-6^. Studies have demonstrated that exposure to e-cigarette aerosols may be associated with airway inflammation, impaired lung function, cardiovascular effects, and nicotine addiction^6^. Furthermore, misconceptions regarding the safety of vaping persist, with many users believing that e-cigarettes emit only water vapor or pose minimal health risks compared with conventional tobacco products^7^.

The use of e-cigarettes has increased considerably in Saudi Arabia. Previous studies conducted among Saudi adults, university students, and young people have reported substantial experimentation and current use of e-cigarettes, with prevalence estimates ranging from approximately 10% to more than 30% depending on the study population and region^2,8-10^. This growing popularity presents a public health concern because vaping may normalize nicotine use, contribute to dual use with conventional tobacco products, and expose users to potential health risks^11,12^. At the same time, evolving regulations and increasing market availability have influenced public perceptions and accessibility of these products^13^.

Sociodemographic determinants play an important role in shaping tobacco-related behaviors, including e-cigarette use. Factors such as gender, age, education level, income, occupation, peer influence, and social norms may influence initiation, frequency of use, perceptions of harm, and smoking cessation behaviors^14-16^. Previous studies have shown that males are generally more likely to use e-cigarettes and to perceive them as less harmful than conventional cigarettes^15,16^. In addition, exposure to family members, friends, and social networks who vape has been identified as a major factor associated with e-cigarette uptake and continued use^14,15^.

Despite the growing body of literature on vaping, a critical knowledge gap remains because official national statistics and dedicated data on adult e-cigarette use in Saudi Arabia are limited. Evidence regarding the sociodemographic determinants, knowledge, perceptions, and patterns of e-cigarette use among Saudi adults remains limited, particularly among users residing in Riyadh. Most existing studies have focused on students, adolescents, or general population samples and have not comprehensively examined the relationship between sociodemographic characteristics, harm perceptions, and vaping behaviors^8-10,15,17,18^. Therefore, this study aimed to investigate the sociodemographic determinants, knowledge, perceptions, and patterns of e-cigarette use among Saudi adult users in Riyadh City, Saudi Arabia. By addressing this gap, our study directly expands the national literature on adult vaping in Saudi Arabia, enables meaningful comparisons with international cross-sectional data, and provides baseline insights to guide local public health strategies and tobacco control policies. By addressing this gap, our study contributes focused exploratory data to the national literature on adult vaping, offers contextual observations for comparison with international literature, and provides preliminary baseline insights to help inform local public health discussions and future nationwide research.

## METHODS

### Study design and participants

A cross-sectional study was conducted among Saudi adult electronic cigarette (e-cigarette) users residing in Riyadh, Saudi Arabia, between January and April 2024. Eligible participants were adults aged ≥18 years who reported current e-cigarette use and resided in Riyadh at the time of data collection. Individuals who did not meet these criteria were excluded.

### Sampling and data collection

Participants were recruited using convenience sampling through social media platforms, including WhatsApp, LinkedIn, and X (formerly Twitter). Data were collected using a self-administered anonymous online questionnaire developed using Google Forms. Ethical approval for this study was obtained from the Institutional Review Board of Alfaisal University, Riyadh, Saudi Arabia (IRB-20271) on 21 January 2024. Electronic informed consent was obtained from all participants before enrollment. At the beginning of the survey, respondents were provided with an information statement describing the study objectives, voluntary participation, and confidentiality measures; it explicitly stated that completing the survey served as their consent to participate and assured them that no identifying information would be collected. All responses were analyzed anonymously.

A minimum sample size of 208 participants was estimated using Cochran’s formula based on the estimated daily smoking rate in Saudi Arabia, a 95% confidence level, and a 5% margin of error. A total of 207 eligible participants completed the survey and were included in the final analysis.

### Survey instrument

The questionnaire was adapted from previously validated instruments, including the Global Youth Tobacco Survey (GYTS)^19^, the International Tobacco Control Youth and Young Adult Tobacco & Vaping Survey^20^, and a survey developed by Alfaraj et al.^15^ for use in Saudi Arabia. The questionnaire was translated from English into Arabic and underwent backward translation into English to ensure conceptual equivalence. Face and content validity were assessed by two public health experts. Before data collection, the Arabic version was pretested among a small group of participants to ensure that the questions were clear and easy to understand. These participants were excluded from the final study sample.

The questionnaire was modified to align with the objectives of the current study and consisted of four domains, including sociodemographic characteristics (gender, age, marital status, education level, income, and occupation), knowledge regarding e-cigarettes and regulations, perceptions and beliefs regarding the safety and health effects of e-cigarettes, and e-cigarette use patterns and behaviors.

### Outcome measures

The primary outcomes included vaping behaviors, knowledge of e-cigarettes, perceptions regarding e-cigarette safety, and harm perception.

A composite harm perception variable was constructed by combining scores across ten survey questions, including: ‘Do you believe that vaping should be banned indoors and in places that ban cigarette smoking like workplaces, restaurants, coffee shops, and cinema theaters?’, ‘Do you believe that vaping is a safer option than nicotine patches or nicotine gum?’, ‘How much do you think people harm themselves when they use e-cigarettes/vape?’, ‘Do you believe that vaping is safer than regular cigarettes and tobacco products?’, ‘Do you think e-cigarettes contain dangerous chemical substances like nicotine, carboxylates, metals, and volatile organic materials in addition to small particles?’, ‘Do you believe that preservatives or flavors used in e-cigarettes are harmful?’, ‘Do you believe that exposing children to e-cigarettes is safe?’, ‘Do you believe that nicotine solutions in cartridges can cause death?’, ‘Does vaping indoors cause harm to non-smokers within the same environment (secondhand smoking) in case of exposure to the aerosol emitted from electronic cigarettes?’, and ‘Do you consider vaping safer for a pregnant woman than traditional tobacco cigarettes?’. Internal consistency reliability gave McDonald’s ω of 0.735 and Cronbach’s of 0.721. No pilot testing was conducted since it was a validated survey.

Scores had a possible range from 0 to 21, indicating no perceived harm to maximally perceived harm. Questions were coded based on their wording direction. Positively worded items, such as ‘Do you believe that nicotine solutions in cartridges can cause death?’, were coded as -1 for No, 0 for Not sure, and +1 for Yes. Conversely, negatively worded items, such as ‘Do you believe that vaping is a safer option than nicotine patches or nicotine gum?’, were reverse-coded so that -1 indicated Yes, 0 indicated Not sure, and +1 indicated No. One item (‘How much do you think people harm themselves when they use e-cigarettes/vape?’) was on a 1 to 4 scale, indicating: no harm, little harm, some harm, a lot of harm. Summing raw responses across these 10 question items produced a scale with a possible range from -8 to 13. The final adjusted harm perception scale used for analysis had 8 added to each participant’s total, giving the 0 to 21 scale.

### Statistical analysis

Data were analyzed using jamovi (version 2.6). Descriptive statistics were used to summarize participant characteristics, vaping behaviors, knowledge, and perceptions. Frequencies and percentages were calculated for categorical variables. All survey items were mandatory; thus, no missing data were present in the dataset.

Associations between categorical variables were assessed using likelihood ratio tests and Mantel-Haenszel tests where appropriate. Ordinal and linear regression models were used to identify sociodemographic determinants associated with vaping frequency and harm perception. Statistical significance was defined as p<0.05. Ordinal multivariable logistic regression was done to evaluate factors associated with vaping frequency (Model A). Multivariable linear regression was done to evaluate factors associated with harm perception (Model B). Model goodness-of-fit was evaluated using McFadden’s R^2^_M_ for Model A and coefficient of determination R^2^ for Model B.

### Potential covariates

Both analyses included (adjusted for) gender, age, and monthly income. Additional variables, such as education level and vaping variables (vaping frequency, vaping led to tobacco, successful tobacco cessation), were added and removed by stepwise removal or addition according to significant changes to R^2^_M_ and R^2^.

Model diagnostics performed for Model B confirmed the absence of influential outliers (maximum Cook’s distance=0.049), and the variance inflation measure for collinearity did not exceed 1.14 (tolerance >0.880). Such diagnostics are not applicable to ordinal logistic regression for Model A.

## RESULTS

### Participant characteristics

A total of 207 Saudi adult e-cigarette users participated in the study. Most participants were male (71.0%) and aged 18–39 years (93.8%). Three-quarters of respondents were single (75.4%), and the majority held a Bachelor’s degree (63.8%). Most participants were employed (79.7%), while 15.0% were students and 5.3% were unemployed. Approximately one-third of participants reported a monthly income below 10000 SAR (Table 1).

**Table 1:** Sociodemographic characteristics of study participants, a cross-sectional study, Riyadh, Saudi Arabia, January–April 2024 (N=207)

*Variable*	*n*	*%*
**Gender**		
Female	60	29.0
Male	147	71.0
**Age** (years)		
18–28	108	52.2
29–39	86	41.6
40–49	11	5.3
50–59	2	1.0
**Marital status**		
Single	156	75.4
Married	42	20.3
Divorced	8	3.9
Widowed	1	0.5
**Education level**		
High school or lower	23	11.1
Diploma	25	12.1
Bachelor’s degree	132	63.8
Postgraduate degree	27	13.0
**Occupation status**		
Employed	165	79.7
Student	31	15.0
Unemployed	11	5.3
**Monthly income** (SAR)		
No income	14	6.8
<10000	72	34.8
10000–15000	45	21.7
15001–20000	27	13.0
>20000	22	10.6
Prefer not to disclose	27	13.0

SAR: 1000 Saudi Riyals about US$260.

Knowledge and perceptions regarding e-cigarettes

Most participants were aware that e-cigarettes are legal in Saudi Arabia (85.0%), while 70.5% correctly identified 18 years as the minimum legal age for e-cigarette use. However, uncertainty regarding regulatory approval remained evident, with only 30.0% reporting that e-cigarettes were authorized by the Saudi Food and Drug Authority (SFDA).

Regarding perceptions of safety, 69.6% believed that e-cigarettes should be banned in locations where conventional smoking is prohibited. Although most participants recognized the potential harms associated with vaping, misconceptions remained common. Approximately 42.5% believed that e-cigarettes are safer than conventional cigarettes, while 13.0% believed that e-cigarettes emit only water vapor. In addition, 74.9% believed that e-cigarettes contain dangerous chemicals, 75.9% considered flavorings and preservatives harmful, and 55.6% believed that secondhand exposure to e-cigarette aerosol may be harmful. Overall, 48.8% perceived e-cigarettes as causing ‘a lot of harm’ (Table 2).

**Table 2:** Knowledge, legality, and perceptions regarding e-cigarettes among study participants, a cross-sectional study, Riyadh, Saudi Arabia, January–April 2024 (N=207)

*Item*	*n*	*%*
**Legality**		
E-cigarettes are legal in Saudi Arabia	176	85.0
Correctly identified minimum legal age (18 years)	146	70.5
**Belief**		
E-cigarettes are authorized by SFDA	62	30.0
Vaping should be banned where smoking is banned	144	69.6
Vaping is safer than nicotine gum/patches	28	13.5
E-cigarettes emit only water vapor	27	13.0
E-cigarettes are safer than conventional cigarettes	88	42.5
E-cigarettes contain dangerous chemicals	155	74.9
Preservatives/flavors are harmful	157	75.9
Exposure to children is unsafe	174	84.1
Vaping may cause death	91	44.0
Secondhand exposure is harmful	115	55.6
Vaping is unsafe during pregnancy	127	61.4
**Harm perception**		
None	4	1.9
Little	24	11.6
Some	78	37.7
A lot	101	48.8

SFDA: Saudi Food and Drug Authority.

### E-cigarette use patterns

Friends and relatives were the primary source of information about e-cigarettes (80.0%). Most participants reported having family members or friends who used e-cigarettes (85.5%) (Table 3).

**Table 3:** E-cigarette use patterns and behaviors among study participants, a cross-sectional study, Riyadh, Saudi Arabia, January–April 2024 (N=207)

*Variable*	*n*	*%*
**Source of information**		
Friends/relatives	166	80.2
Social media	18	8.7
Other	19	9.2
Television/newspapers	4	1.9
Family/friends who vape	177	85.5
**Daily vaping frequency**		
>1	177	85.5
≤1	30	14.5
**Vaping episodes per day**		
0–4	40	19.3
5–9	38	18.4
10–14	28	13.5
15–19	21	10.1
20–29	10	4.8
≥30	70	33.8
**Purchase source**		
Tobacco/shisha shop	92	44.4
E-cigarette shop	89	43.0
Internet	13	6.3
Other	13	6.3
**Smoking status**		
Current tobacco smoking	66	31.9
Previous tobacco smoking	54	26.1
Never smoked tobacco	87	42.0
Successful tobacco cessation through vaping	79	38.2
Reduced tobacco use through vaping	36	17.4
Vaping-related ED visit/hospitalization	17	8.2

ED: emergency department.

The majority of participants reported vaping more than once daily (85.5%), and approximately one-third (33.8%) reported vaping ≥30 times per day. More than half of respondents (51.2%) indicated that they vaped in all situations, while 65.2% reported vaping both indoors and outdoors. Tobacco and shisha shops (44.4%) and specialized e-cigarette shops (43.0%) were the most common purchasing locations.

Regarding smoking behavior, 31.9% of participants currently smoked conventional tobacco products, whereas 26.1% reported previous tobacco use. Approximately 38.2% reported successfully quitting conventional tobacco through vaping, while 17.4% reported reduced tobacco use. Only 8.2% reported a vaping-related emergency department visit or hospitalization.

The most frequently reported reasons for vaping were curiosity or trying something new (40.0%), enjoyment (35.7%), flavor preferences (31.4%), nicotine delivery (28.5%), and smoking cessation (27.5%).

### Associations between sociodemographic factors and vaping behavior

Significant associations were observed between gender and vaping situations (LR χ^2^(3)=8.215, p=0.042), occupation and vaping situations (LR χ^2^(6)=14.34, p=0.026), and age and vaping location (LR χ^2^(6)=12.63, p=0.049) (Table 4).

**Table 4:** Associations between sociodemographic factors and vaping behavior among study participants, a cross-sectional study, Riyadh, Saudi Arabia, January–April 2024 (N=207)

*Factors*	*Past 30 days use situations of e-cigarettes*				
	*Alone* *n (%)*	*At Work* *n (%)*	*Everywhere* *n (%)*	*With friends* *n (%)*	*Total n*
**Gender**					
Female	20 (9.7)	1 (0.5)	27 (13.0)	12 (5.8)	60
Male	25 (12.1)	10 (4.8)	79 (38.2)	33 (15.9)	147
Total	45 (21.7)	11 (5.3)	106 (51.2)	45 (21.7)	207
Statistical tests	LR χ²(3)=8.215, **p=0.042**				
**Occupation status**					
Employed	29 (14.0)	9 (4.3)	86 (41.5)	41 (19.8)	165
Student	13 (6.3)	1 (0.5)	13 (6.3)	4 (1.9)	31
Unemployed	3 (1.5)	1 (0.5)	7 (3.4)	0 (0)	11
Total	45 (21.7)	11 (5.3)	106 (51.2)	45 (21.7)	207
Statistical tests	LR χ²(6)=14.34, **p=0.026**				
	***Vaping location***				
	***Indoors n (%)***	***Outdoors n (%)***	***Indoors and Outdoors n (%)***		***Total n***
**Age** (years)					
18–28	10 (4.8)	35 (16.9)	63 (30.4)		108
29–39	8 (3.9)	13 (6.3)	65 (31.4)		86
40–49	0 (0)	5 (2.4)	6 (2.9)		11
50–59	0 (0)	1 (0.5)	1 (0.5)		2
Total	18 (8.7)	54 (26.1)	135 (65.2)		207
Statistical tests	LR χ²(6)=12.63, **p=0.049**				
	***Is vaping safer than regular cigarettes and tobacco products?***				
	***No n (%)***	***Not sure n (%)***	***Yes n (%)***		***Total n***
**Gender**					
Female	33 (15.9)	4 (1.9)	23 (11.1)		60
Male	53 (25.6)	29 (14.0)	65 (31.4)		147
Total	86 (41.5)	33 (15.9)	88 (42.5)		207
Statistical tests	LR χ²(2)=9.226, **p=0.010**				
	***Is only water vapor emitted from electronic cigarettes?***				
	***No n (%)***	***Not sure n (%)***	***Yes n (%)***		***Total n***
**Gender**					
Female	44 (73.3)	12 (20.0)	4 (6.7)		60
Male	78 (53.1)	46 (31.3)	23 (15.7)		147
Total	122 (59)	58 (28)	27 (13)		207
Statistical tests	LR χ²(2)=7.920, **p=0.019**				

Associations were assessed using the likelihood ratio LR χ² (df) test, with degrees of freedom df. p<0.05 was considered statistically significant.

Gender was also significantly associated with perceptions of e-cigarette safety compared with conventional cigarettes (LR χ^2^(2)=9.226, p=0.010). Male participants were more likely to perceive e-cigarettes as safer than conventional cigarettes, whereas female participants were more likely to disagree. In addition, gender was significantly associated with beliefs regarding e-cigarette emissions (LR χ^2^(2)=7.920, p=0.019), with males more likely to believe that e-cigarettes emit only water vapor (Table 4).

### Factors associated with vaping frequency

Vaping frequency was defined based on responses to ‘How frequently do you vape?’, categorized as more than once a day, once a day, once or twice a week, once or twice a month, and fewer than once a month. Multivariable ordinal logistic regression analysis was adjusted for gender, age, education level, occupation, and income (R^2^_M_=0.094). Male participants on average reported significantly higher vaping frequency compared with females (B=1.870; 95% CI: 0.913 –2.891; p<0.001). No other statistically significant associations were observed (Table 5).

**Table 5:** Multivariable regression analyses of vaping frequency and harm perception among study participants, a cross-sectional study, Riyadh, Saudi Arabia, January–April 2024 (N=207)

*Variable*	*B*	*95% CI*	*p*
**Model A: Factors associated with vaping frequency**			
Male gender	1.870	0.913–2.891	<0.001
Age (years)	0.554	-0.355–1.594	0.202
Education level	-0.316	-1.088–0.374	0.367
Student vs employed	-0.005	-1.313–1.405	0.931
Income	0.057	-0.417–0.564	0.681
**Model B: Factors associated with harm perception**			
Male gender	-1.583	-2.947 – -0.219	0.023
Age (years)	-0.774	-1.776–0.227	0.129
Vaping frequency	0.145	-0.523–0.805	0.669
Vaping led to tobacco initiation	0.396	-1.013–1.805	0.580
Successful tobacco cessation through vaping	-2.802	-4.496 – -1.108	0.001
Monthly income	0.293	-0.262–0.847	0.299

B: log odds ratio, unstandardized regression coefficient. CI: confidence interval. Vaping frequency was defined based on responses to ‘How frequently do you vape?’, categorized as more than once a day, once a day, once or twice a week, once or twice a month, and fewer than once a month. Harm perception was defined and measured using a combined score calculated from 10 survey items, where higher scores reflect a greater perception of harm associated with e-cigarettes. p<0.05 was considered statistically significant. Reference categories were female for gender, employed for occupation, no for vaping leading to tobacco initiation, and no change for successful tobacco cessation through vaping. Model fit for Model A: McFadden’s R_M_^2^=0.094, and Model B: R^2^=0.142.

### Factors associated with harm perception

For harm perception, it was defined and measured using a composite score derived from 10 survey items, with higher scores indicating greater perceived harm of e-cigarettes. Multivariable linear regression analysis was adjusted for gender, age, vaping frequency, vaping leading to tobacco initiation, successful tobacco cessation through vaping, and monthly income (R^2^=0.142). Male participants reported significantly lower harm perception scores than females (B= -1.583; 95% CI: -2.947 – -0.219; p=0.023). In addition, participants who reported successfully quitting conventional tobacco through vaping had significantly lower harm perception scores than those who reported no change in tobacco use (B= -2.802; 95% CI: -4.496 – -1.108; p=0.001). No significant associations were observed for age, vaping frequency, income, or vaping leading to tobacco initiation (Table 5).

## DISCUSSION

This study investigated the sociodemographic determinants, knowledge, perceptions, and patterns of e-cigarette use among Saudi adult users in Riyadh. Four principal findings emerged. First, male participants reported significantly higher vaping frequency and lower harm perception compared with females. Second, misconceptions regarding e-cigarette safety remained prevalent despite generally high awareness of vaping regulations. Third, social influences appeared to be strongly associated with vaping behavior, with friends and relatives representing the primary source of information regarding e-cigarettes. Finally, participants who reported successfully quitting conventional tobacco through vaping perceived e-cigarettes as less harmful than those who did not report cessation success.

Knowledge and perceptions regarding e-cigarettes

Although most participants were aware that e-cigarettes are legal in Saudi Arabia and correctly identified the minimum legal age for use, substantial misconceptions regarding e-cigarette safety persisted. More than two-fifths of participants believed that e-cigarettes are safer than conventional cigarettes, and 13% believed that e-cigarettes emit only water vapor. These findings are concerning because inaccurate perceptions of reduced harm may contribute to increased uptake and continued use of e-cigarettes.

Our findings are consistent with previous Saudi studies reporting widespread misconceptions regarding e-cigarette safety. Althobaiti and Mahfouz^2^ reported high levels of experimentation and favorable perceptions of e-cigarettes among Saudi adults. Similarly, Hegazy et al.^17^ found considerable uncertainty among Saudi adults regarding the safety and regulatory status of e-cigarettes. Together, these findings suggest that increasing availability and social acceptance of e-cigarettes may not necessarily be accompanied by an accurate understanding of their health risks.

Despite these misconceptions, most participants recognized that e-cigarettes contain dangerous chemicals and believed that exposure to children and pregnant women may be harmful. These findings are encouraging and align with growing scientific evidence demonstrating that e-cigarette aerosols contain nicotine, toxic compounds, heavy metals, and ultrafine particles that may adversely affect respiratory and cardiovascular health^4,6^. However, the coexistence of awareness regarding harmful constituents and the belief that e-cigarettes are safer than conventional cigarettes, highlights the complexity of public perceptions surrounding vaping.

### Vaping behaviors and social influences

The present study demonstrated a high prevalence of frequent vaping among participants, with most respondents reporting vaping more than once daily. Curiosity, enjoyment, flavor preferences, nicotine delivery, and smoking cessation were the most commonly reported motivations for use. These findings are broadly consistent with previous studies conducted in Saudi Arabia and internationally, which have identified curiosity, social influence, flavors, and smoking cessation as major drivers of e-cigarette use^18,21^.

A notable finding was the strong association with social networks. Most participants reported learning about e-cigarettes through friends or relatives, and the majority indicated that family members or friends also used e-cigarettes. Similar findings have been reported by Alfaraj et al.^15^ and Khateeb et al.^22^, suggesting that peer influence remains an important determinant of vaping behavior within Saudi communities. Social acceptance of vaping may contribute to normalization of nicotine use and facilitate initiation, particularly among younger adults.

The majority of participants purchased e-cigarettes from tobacco and shisha shops or specialized vape stores, which is similar to the findings reported by Alluhaidan et al.^23^, reflecting the increasing accessibility of vaping products within Saudi Arabia.

### Gender differences in vaping behavior and harm perception

Gender emerged as the most consistent factor associated with vaping behavior in the present study. Male participants reported significantly higher vaping frequency than females and were more likely to perceive e-cigarettes as safer than conventional cigarettes. Furthermore, males were more likely to believe that e-cigarettes emit only water vapor and demonstrated significantly lower harm perception scores.

These findings are consistent with previous research. Dahal et al.^24^ reported that e-cigarette use was more common among males than females, while Wamamili et al.^16^ found that men were more likely to perceive e-cigarettes as less harmful than conventional cigarettes. Similarly, Ceasar et al.^25^ observed that male participants were more likely to believe that e-cigarettes were safer than traditional cigarettes.

Several explanations may account for these gender differences. Men generally exhibit higher rates of tobacco and nicotine use globally and may be more likely to engage in risk-taking behaviors. In addition, marketing strategies frequently portray vaping as a socially acceptable or modern alternative to smoking, which could also reflect social desirability bias, as male respondents might downplay health risks or adjust their responses to fit social expectations or masculine norms.

### Harm perception and smoking cessation

Participants who reported successfully quitting conventional tobacco through vaping demonstrated significantly lower harm perception scores than those who reported no change in tobacco use. This finding suggests that individuals who perceive vaping as an effective smoking cessation tool may also view it as less harmful.

Previous studies have reported mixed findings regarding the relationship between harm perceptions and smoking cessation behaviors. Lin et al.^26^ found that perceptions regarding the relative harm of nicotine products were associated with quitting intentions among cigarette smokers. Individuals who believe that e-cigarettes are less harmful may be more willing to adopt them as alternatives to combustible tobacco products^26^. While some evidence suggests that e-cigarettes may contribute to smoking reduction or cessation among certain users, uncertainty remains regarding their long-term effectiveness and health consequences. These mixed findings across studies may relate to differences in population size, local cultural context, national tobacco control policies, and study design. For example, while Lin et al. analyzed data from a nationally representative longitudinal cohort in the US, our study examined e-cigarette users in a single city (Riyadh). Differences in education level, age, social environment, national vaping regulations, and regional vaping habits across these study groups can correspond with varying health awareness and risk perceptions. Ultimately, evaluating behaviors across longitudinal cohort tracking versus single-point cross-sectional observation can explain why observed associations differ from one study to another.

### Limitations

Most participants in the study were males. Furthermore, the use of a convenience sample and a self-administered online questionnaire may have introduced response bias, recall bias, and social desirability bias, which limits the generalizability of the results to the entire population of Saudi e-cigarette users. The study’s cross-sectional nature introduces temporal ambiguity and selection bias, as it captures a single point in time and does not allow for causal inferences. Additionally, conducting the study in only one city may restrict the findings to that location’s specific demographic and social factors. Moreover, the sample size was small due to the restricted timeframe available to complete this research, which may compromise the robustness of the results. Furthermore, despite adjusting for key variables, the possibility of residual confounding from unmeasured factors remains. Finally, no pre-testing was performed for the 10-item composite harm perception variable. These constraints underscore the need for further research with larger, nationwide samples and validated instruments.

## CONCLUSIONS

This study provided crucial insights into the sociodemographic determinants associated with e-cigarette use among Saudi adult users in Riyadh City. The study investigated the sociodemographic determinants and found that gender, age, and current occupation were associated with e-cigarette use. Additionally, the study showed statistically significant associations between gender, age, and occupation related to vaping behaviors. Males were more likely to vape frequently compared to females. Age also was associated with the locations where individuals vaped, while current occupation was linked to the situations in which vaping occurred.

Furthermore, the study measured various perceptions of Saudi e-cigarette smokers and showed a statistically significant association between gender and belief about electronic cigarette emissions, gender and perception of the perceived safety of e-cigarettes compared to cigarettes, and a correlation between age and the belief that e-cigarettes are legal in Saudi Arabia. The study also assessed patterns of e-cigarette use and identified several significant relationships. It found a notable connection between age and vaping locations, as well as a significant relationship between current occupation and the situations in which individuals vaped. Additionally, there was a relationship between the frequency of vaping and gender.

When testing e-cigarette users’ knowledge about e-cigarettes, the study found a significant correlation between respondents’ age and the belief that e-cigarettes are legal in Saudi Arabia, and their awareness of the presence of dangerous chemicals in e-cigarettes, as well as age and the potential harm associated with preservatives and flavors, and a correlation between the frequency of vaping and belief about the legality of e-cigarettes. Moreover, while investigating harm perception, the study found that individuals who successfully quit tobacco products through vaping tended to have a lower perception of harm, and males generally had lower harm perception scores than females.

Ultimately, this study acknowledges key limitations, including its cross-sectional design and the use of a convenience sample, which may limit the generalizability of the findings. Future studies should further investigate these associations and explore e-cigarette use behaviors among this population. Continued surveillance and longitudinal research are warranted to monitor evolving patterns of e-cigarette use and inform tobacco control policies in Saudi Arabia.

## Data Availability

The data supporting this research are available from the authors on reasonable request.
